# A Case of Angiosarcoma Arising in Trunk of the Right Pulmonary Artery Clinically Simulating Pulmonary Thromboembolism

**DOI:** 10.4021/wjon467w

**Published:** 2012-07-05

**Authors:** Yasuhiro Nakamura, Toru Shimizu, Yoshihiro Fukumoto, Koichiro Sugimura, Shigemi Ito, Fumiyoshi Fujishima, Mariko Oikawa, Mika Watanabe, Hiroaki Shimokawa, Hironobu Sasano

**Affiliations:** a Department of Pathology, Tohoku University Graduate School of Medicine, Sendai, Japan; b Department of Cardiovascular medicine, Tohoku University Graduate School of Medicine, Sendai, Japan; c Devision of Pathology, Miyagi Cancer Center, Natori, Japan; d Division of Oral Pathology, Department of Oral Medicine and Surgery, Tohoku University Graduate School of Dentistry, Sendai, Japan

**Keywords:** Angiosarcoma, Pulmonary artery, FDG-PET, Immunohistochemistry

## Abstract

Angiosarcoma arising in the pulmonary artery is extremely rare. We herein report a case of angiosarcoma arising in the pulmonary artery trunk of 71 year-old woman. This case was clinically diagnosed as pulmonary thromboembolism but angiosarcoma of the pulmonary artery should be considered as the differential diagnosis of unusual clinical manifestations of pulmonary thromboembolism because of the extremely poor prognosis of the lesion.

## Introduction

Angiosarcoma of pulmonary artery is very rare [1-15]. In addition, this tumor has been previously postulated to be indistinguishable from thromboembolism of the pulmonary artery [13, 15]. We herein report a case of 71 year-old woman initially diagnosed as pulmonary thromboembolism and proven to be pulmonary angiosarcoma arising in the pulmonary artery trunk by autopsy.

## Case Report

A 71-year-old woman with one month history of progressive shortness of breath, cough and atypical chest pain was hospitalized and diagnosed as pulmonary thromboembolism by computed tomography (CT) scan. She was then treated with heparin followed by warfarin, which mildly improved her symptoms but subsequent CT scan did not reveal any improvement. The patient was subsequently diagnosed as chronic thromboembolic pulmonary hypertension (CTEPH) and discharged. In 4 months later, she developed sudden-onset of dyspnea, high fever and a large pericardial effusion on transthoratic echocardiography (TTE). The patient was readmitted to the intensive care unit of the hospital. Subseunet pericardiocenthesis revealed serosanginous pericardial effusion negative for infection and malignancy and she was subsequently referred to Tohoku University Hospital, Sendai, Japan.

Physical examination on admission revealed a loud P2 and a Grade III/IV systolic murmur, jugular vein dilatation and hepatomegaly. Her blood pressure was 90/60 mmHg, pulse rate 100 beats/min, body temperature 37.2 °C, SpO2 98% with a 2 L/min O2 and she had a respiration rate of 20 breaths/min (NYHA III). The electrocardiogram demonstrataed sinus tachycardia (110 beats/min), P-wave pulmonale, T wave inversion on the right precordial leads, and incomplete right bundle block implying a pressure and volume overload of the right ventricle. Subsequent chest X-ray revealed cardiomegaly and bilateral pleural effusion with cardiothoracic ratio of 70%. The laboratory investigation indicated anemia (hemoglobin 8.6 g/dl), heart failure (brain natriuteric peptide 183 pg/ml), and suspected embolism (D-dimer 7.7 µg/ml).

**Figure 1:**
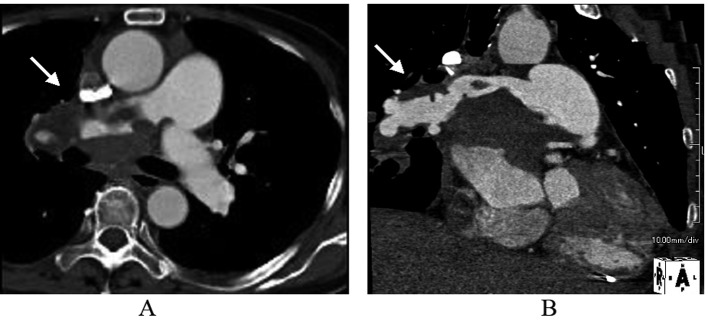
The image of Contrast-enhanced computed tomography scan. Early arterial phase showed massive filling defects in the proximal portion of right pulmonary artery and around the left atrium in axial (A) and sagittal (B) views.

**Figure 2:**
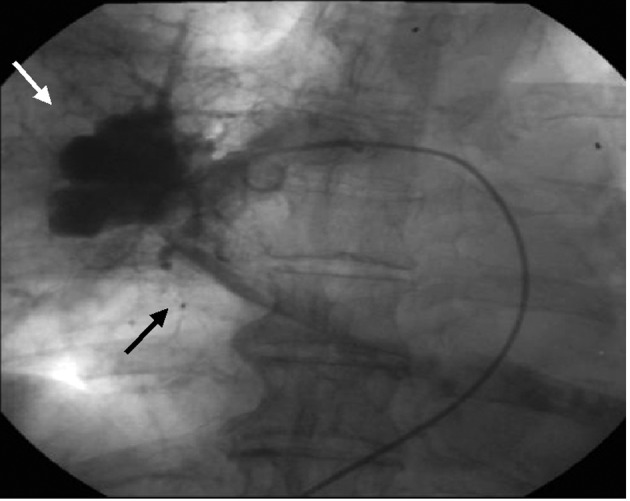
The image of right pulmonary angiography. Vascularized mass (white arrow) with a fistula from the right pulmonary artery to the left atrium (black arrow).

**Figure 3:**
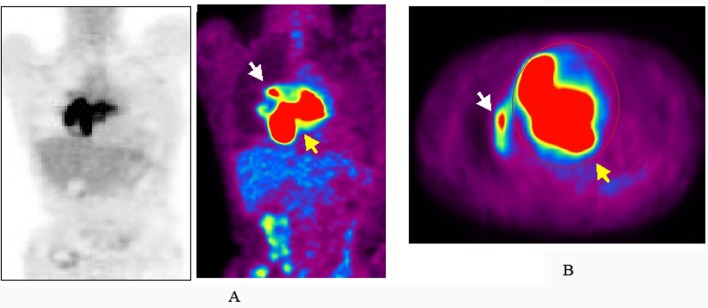
The image of maximum intensity projection image of 18-fluorodeoxyglucose positron emission tomography (FDG-PET). Increased uptake of FDG at the area of the right pulmonary artery (white arrow) and the bilateral atria (yellow arrow) in coronal (A) and axial (B) views.

TTE revealed dilatation of the main pulmonary trunk and high systolic pressure gradient across the tricuspid valve of 49 mmHg that is consistent with pulmonary hypertension. There was a thrombus-like mass in the right pulmonary artery and around the left atrium. CT scan similarly revealed a large mass in the proximal portion of right pulmonary artery and around the left atrium, but no abnormal mass detected in the lung and other organs (Fig. 1). However, lung scintigraphy demonstrated little visualization of the right lung on technetium-99m macroaggregated albumin (99mTc-MAA) perfusion scan. Ventilation scan with krypton-81m (81mKr) gas was normal (ventilation-perfusion mismatch). Cardiac catheterization demonstrated mean pulmonary artery pressure of 40 mmHg that indicated pulmonary hypertension. Right pulmonary angiography demonstrated vascularized mass with a fistula from the right pulmonary artery to the left atrium (Fig. 2). To confirm this suspected malignant features and the extent of disease, 18-fluorodeoxyglucose positron emission tomography (FDG-PET) was performed. FDG-PET revealed a large mass at the area of the right pulmonary artery and the bilateral atira with intense uptake of FDG. The maximum standard uptake value (SUV_max_) was 25.2. No other undue hypermetabolic lesions were noted elsewhere (Fig. 3).

On hospital day 24, a CT-guided transthoracic biopsy of the pericardial mass was performed. A histological diagnosis was a high-grade sarcoma, pleomorphic or spindle cell type and subsequent immunohistochemical findings consistent with angiosarcoma. She consequently underwent chemotherapy with paclitaxel but on hospital day 55, the patient demised due to resistant right heart failure and consecutive respiratory failure during first-line chemotherapy.

**Figure 4:**
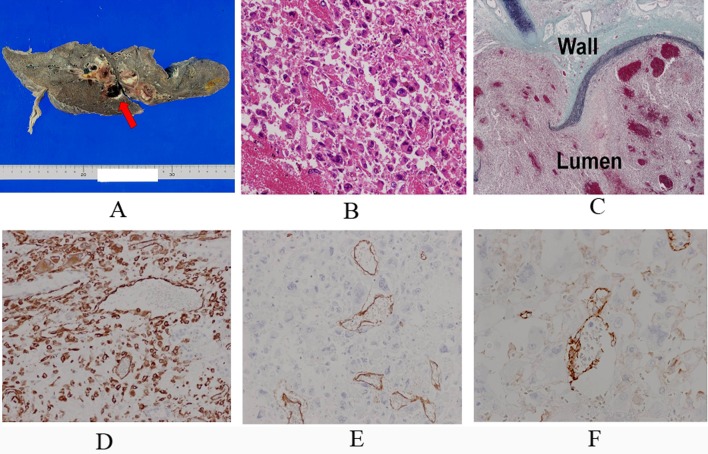
A. Macroscopical finding showed that the right main trunk and its second branched segments of the pulmonary artery were completely occluded by the tumor and thrombus. A red arrow indicates the right pulmonary artery. B. Hematoxilin and eosin staining showed that the tumor cells were composed of pleomorphic cells which had large and vesicular nuclei with prominent nucleori and abundant eosinophilic cytoplasm. C. Elastica and masson staining showed the destruction of the wall of the right pulmonary artery by the tumor cells. ‘Wall’ indicates the wall of the right main trunk of the pulmonary artery, and ‘Lumen’ indicates its lumen. D. D, E and F: Immunohistochemical findings showed the tumor cells were diffusely positive for vimentin (D), focally positive for CD31 (E) and CD34 (F) in the vascular channel area.

An autopsy was performed. The tumor measured 20 x 8 cm in greatest diameter and occupied the upper pericardial space surrounding the pulmonary artery with direct invasion into both atriums and right lung (Fig. 4). The inner surface of the right pulmonary artery appeared irregular, and its second branched segment was completely occluded by the presence of tumor. Large coagulative thrombus was impacted in both atrioventricular outflows. Marked congestion and edema with focal hemorrhage were detected in bilateral lungs.

Histologically, the tumor was derived from the surface of the right pulmonary and was composed of pleomorphic cells harboring large and vesicular nuclei with prominent nucleoli and abundant eosinophilic cytoplasm (Fig. 4). Numerous mitoses and atypical mitoses were detected. Foci of hemorrhage and necrosis were also detected. Tumor cells focally formed vascular channels containing red blood cells. Immunohistochemically, the tumor cells were diffusely positive for vimentin, focally positive for CD31 and CD34 (Fig. 4) and negative for AE1/AE3, factor VIII-related antigen, a-SMA, S-100, desmin, HHF-35, myoD1 and D2-40 (data not shown). MIB-index of tumor cells was approximately 15% (Fig. 4). The tumor was finally diagnosed as angiosarcoma originating in the trunk of the right pulmonary artery. Metastatic lesion was not detected.

## Discussion

The incidence of pulmonary angiosarcoma is reported to be even only 3.6% among pulmonary artery sarcomas [2]. Huo et al reviewed 11 cases of angiosarcoma derived from pulmonary artery reported in the English literature [1] and reported as follows: the mean age of the 11 patients was 55 years (range, 38 - 69 years), and 5 were men and 2 were women among the 7 patients of known sex [1]. In the majority of cases (n = 8), the main trunk, right and/or left pulmonary artery were involved among the 10 cases of known tumor localization [1]. There was one case where only the right pulmonary artery was involved, and lung involvement was reported in 5 of 11 cases as in our case [1]. In addition, both the right and left atrium were involved by the tumor in our case, which has not been reported in any of the previously reported cases.

Echocardiography is a fundamental and useful diagnostic tool for pulmonary artery sarcoma. Contrast-enhanced CT scan or magnetic resonance imaging with gadolinium enhancement could be more beneficial for diagnosis of pulmonary artery sarcoma, which could provide the additional information regarding the size, location, attachment, and local extent of the tumor. Recently, FDG-PET has been reported to be useful for evaluation of the local invasion and distant metastasis of malignant cardiac tumors and for monitoring the response to therapy [16-19]. In addition, Ito et al reported FDG-PET can distinguish pulmonary artery sarcoma from venous pulmonary thromboembolism based on the FDG uptake with SUV_max_ (7.63 ± 2.21 vs. 2.31 ± 0.41) [18]. In our case, the high FDG uptake with SUV_max_ of 25.2 has highly suspected pulmonary artery sarcoma, but not thromboembolism.

The tumor in our case was diagnosed as angiosarcoma at autopsy. The histological features of our present case demonstrated a high-grade spindle and pleomorphic cell with vascular channels. Immunohistochemically, the tumor cells were diffusely positive for vimentin, focally positive for CD31 and CD34 in our case, which are consistent with immunohistochemical features of angiosarcoma [14]. While cytokeratin was negative for the tumor in our case, previous studies demonstrated cytokeratin immunoreactivity in pulmonary angiosarcoma [4, 14].

The most common presenting symptoms are known to be non-specific, including cough, dyspnea, chest pain and intermittent hemoptysis [1, 13]. Therefore, several previous cases were initially diagnosed as pulmonary thromboembolism [8, 13, 15]. It has been postulated that pulmonary artery angiosarcoma should be included in the differential diagnosis of acute or chronic pulmonary thromboembolism at initial diagnosis, since the prognosis of pulmonary artery sarcoma in considered extremely poor. However, Engelke et al. postulated that pulmonary thromboembolism caused by primary pulmonary malignancy is difficult for therapy since thrombolysis or therapeutic anticoagulation are usually contraindicated in patients with underlying pulmonary malignancy to prevent serious bleeding complications [15]. In addition, biopsy could contribute greatly to the differential diagnosis between primary parenchymal and pulmonary arterial sarcomatous tissues [15]. It is postulated that pulmonary artery sarcoma is considered a disease of unilateral pulmonary artery involvement, whereas pulmonary embolism seems comparatively to be a disease of bilateral pulmonary artery involvement as seen in our case [20]. Therefore, in our pesent case, pulmonary artery angiosarcoma should have been included at the initial diagnosis.
